# Combination of EGFR Inhibitor Lapatinib and MET Inhibitor Foretinib Inhibits Migration of Triple Negative Breast Cancer Cell Lines

**DOI:** 10.3390/cancers10090335

**Published:** 2018-09-17

**Authors:** Aleksandra Simiczyjew, Ewelina Dratkiewicz, Marleen Van Troys, Christophe Ampe, Ilona Styczeń, Dorota Nowak

**Affiliations:** 1 Department of Cell Pathology, Faculty of Biotechnology, University of Wroclaw, Joliot-Curie 14a, 50-383 Wroclaw, Poland; ewelina.dratkiewicz@uwr.edu.pl (E.D.); ilona.styczen@uwr.edu.pl (I.S.); dorota.nowak@uwr.edu.pl (D.N.); 2 Department of Biochemistry, Faculty of Medicine and Health Sciences, Ghent University, Albert Baertsoenkaai 3, B-9000 Ghent, Belgium; Leen.VanTroys@UGent.be (M.V.T.); Christophe.Ampe@UGent.be (C.A.)

**Keywords:** receptor tyrosine kinases, EGFR, MET, RTK inhibitors, triple negative breast cancer

## Abstract

Triple-negative breast cancer (TNBC) is the most challenging subtype to treat due to the lack of estrogen receptor, progesterone receptor, and HER2 expression, which excludes the usage of directed targeted therapy against them. Promising therapeutic targets are the hepatocyte growth factor receptor (MET) and epidermal growth factor receptor (EGFR), which expression is frequently elevated in TNBC. Inhibitors of these receptors used as monotherapy are often ineffective. Due to that, we studied the efficacy of combined therapy targeting MET and EGFR simultaneously. Two TNBC cell lines were treated with lapatinib (a dual EGFR and HER2 inhibitor), foretinib (a MET inhibitor), or a combination of the two. After the inhibitors treatment, we verified the cell viability (XTT assay), distribution of the cell cycle phases, the activation of signaling pathways (Western blotting), distribution of invadopodia, fluorescent gelatin digestion (immunofluorescence), and the invasion capacity of cells. A combination of foretinib and lapatinib effectively reduced the viability of examined cells, led to G2/M arrest and reduction of pAKT. There was also a decreasein number of invadopodia formed by cells, their ability to digest gelatin and reduction of cells migration/invasion capacity. Therapy targeting of both EGFR and MET receptors was much more effective against tested cells than monotherapy. We selected a combination of drugs that could be successfully used against this breast cancer subtype.

## 1. Introduction

Triple-negative breast cancer (TNBC) accounts for 15% of breast cancer cases and it is associated with an advanced stage at the time of diagnosis and early occurrence of distant metastases. It also has the poorest survival outcome of all breast cancer subtypes [1]. The lack of expression of the HER2 (receptor tyrosine-protein kinase erbB-2), the estrogen receptor (ER), and the progesterone receptor (PR) characterizes the TNBC cells [1,2]. Due to the lack of these proteins and the possibility of using targeted therapy against these receptors, chemotherapy is currently the only systemic treatment method available for TNBC patients [1,3]. Therefore, TNBC is the most challenging breast cancer subtype in terms of treatment. It is believed that receptor tyrosine kinases (RTKs) particularly the hepatocyte growth factor receptor (MET) and epidermal growth factor receptor (EGFR) are promising therapeutic targets due to their elevated expression in multiple TNBC subtypes [2].

It was shown that EGFR is overexpressed in approximately 50% of TNBC cases and its high expression correlates with a poor prognosis [4,5,6,7]. EGFR is a tyrosine kinase receptor involved in cell growth and survival [8,9]. It belongs to the membrane anchored receptor tyrosine kinase ERBB/HER family consisting of EGFR, HER2, HER3, and HER4 [10]. Ligand activation of EGFR results in homo- and hetero-dimerization with other family members. This dimerization enables EGFR auto-phosphorylation, which results in the recruitment of signaling proteins to the receptor [8,9]. Aberrant activation of EGFR in cancer cells as a result of gene copy number amplification, protein overproduction, or point mutations leads to unregulated proliferation, malignant transformation, invasion, metastasis, and resistance to apoptosis [5,10]. EGFR overexpression correlates with a loss of estrogen responsiveness and a poor prognosis [11]. However, therapies directed only against EGFR based on inhibitors or monoclonal antibodies were not effective in TNBC [3].

Receptor tyrosine kinase MET of which the ligand is a hepatocyte growth factor is also highly expressed in TNBC [12]. It is overexpressed in 20% to 30% of breast cancer cases and appears to be associated with a worse prognosis [13]. MET can be activated through ligand-dependent and ligand-independent mechanisms [9]. Ligand-dependent activation of this receptor occurs in the mammary gland where stromal cells such as fibroblasts produce HGF [14]. Ligand-independent activation of MET occurs through MET mutation, constitutive dimerization of the receptor as a result of overexpression, crosstalk with other membrane receptors including the EGFR, or the loss of negative regulators [9,15,16,17]. Excessive MET activation promotes the growth, survival, and migration of cancer cells [17]. MET signaling, which is similar to EGFR, activates a number of downstream effectors such as AKT, extracellular signal-related kinase (ERK), phosphoinositide 3-kinase, RAS, and SRC [16,18]. MET expression correlates positively with EGFR expression in basal-type breast cancers [9]. Of particular interest is the fact that MET gene amplification and overexpression leads to resistance to anti-EGFR therapies in non-small cell lung cancer [19] and in brain tumors through receptor co-activation with EGFR [20].

In TNBC, even in the presence of EGFR inhibitors, cancer cells exhibit perpetual phosphorylation of this receptor, which correlates with the resistance to tyrosine kinase inhibitors (TKI) [2,21]. This resistance may be related to MET–EGFR crosstalk, which was reported to be involved in therapeutic resistance to EGFR inhibitors in colon and lung cancers [19,22]. EGFR and MET interact both physically and functionally in a way independent of both receptors’ kinase activities and this interplay can promote resistance to EGFR TKI via the constitutive phosphorylation of MET [9]. In this scenario, we report on the influence of a mix of selected EGFR and MET inhibitors on TNBC cell viability, cytoskeleton organization, and invasion in vitro. The aim of our study is to support new potential therapeutic strategies for triple negative breast cancer treatment.

## 2. Results

### 2.1. The Combination of EGFR and MET Inhibition Has Synergistic Cytotoxic and Cytostatic Effects on Triple-Negative Breast Cancer Cell Lines

TNBC often displays overexpression of EGFR and MET [4,5,12] and the crosstalk between these receptors has been implicated in therapeutic resistance to EGFR inhibitors. We decided to test the influence of combining inhibitors of EGFR (lapatinib) and MET (foretinib) on the viability and invasive capacity of two selected TNBC cell lines: MDA-MB-231 and BT549.

**Figure 1:**
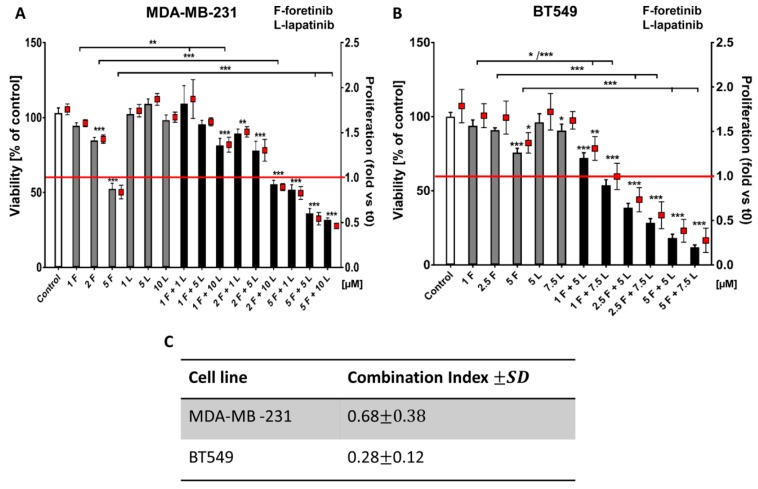
The effects of foretinib, lapatinib, and their combination on TNBC cell line viability and proliferation were measured using XTT assay (see the methods section). Cells were seeded on a layer of polymerized collagen. EGF and HGF was present in all conditions. Viability and proliferation of (**A**) MDA-MB-231 cells and (**B**) BT549 cells after 24 h of incubation with the indicated concentrations of inhibitors (in µM) were compared to those of control cells (percentage of viable cells versus control with a control set to 100 or proliferation-based fold change versus control). Results expressed as the mean ± SD are based on at least three independent experiments. The statistical significance was assessed versus that of the control. The significance level was set at *p* ≤ 0.05 (*), *p* ≤ 0.01 (**), or *p* ≤ 0.001 (***). (**C**) The combination index (CI) after 24 h of drug treatment was determined. Drug combinations in which CIs were <1.0 were considered as synergistic.

**Figure A1:**
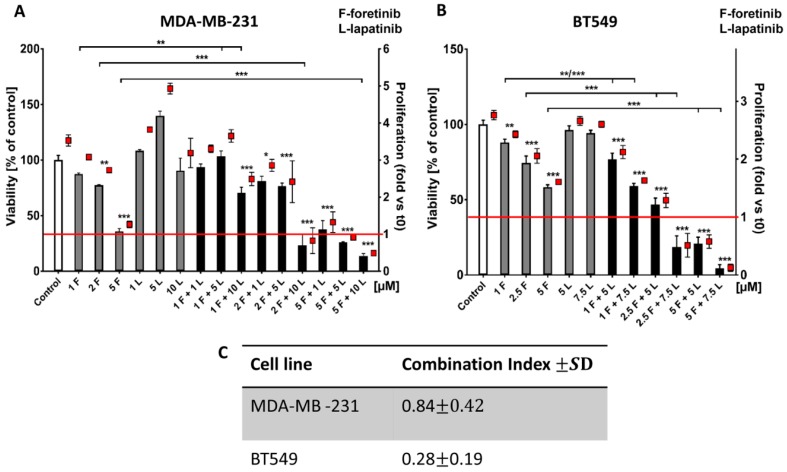
Effects of foretinib, lapatinib, and their combination on TNBC cell line viability and proliferation. Cell viability and proliferation was measured using an XTT assay (as discussed in the methods section). Cells were seeded on a layer of polymerized collagen. EGF and HGF was present in all conditions. Viability and proliferation of (**A**) MDA-MB-231 cells and (**B**) BT549 cells after 48 h incubation with the indicated concentrations of inhibitors (in µM) were compared to those of control cells (percentage of viable cells versus control cells with the control set to 100 or proliferation-based fold change versus the control). Results, which are expressed as the mean ± SD, are based on at least three independent experiments. The statistical significance was assessed versus the control. The significance level was set at *p* ≤ 0.05 (*), *p* ≤ 0.01 (**), or *p* ≤ 0.001 (***). (**C**) The combination index after 48 h of drug treatment was determined. Drug combinations in which CIs were <1.0 were considered to be synergistic.

We first evaluated the cytotoxic effects and the influence on proliferation of the single agents and their mix using an XTT assay. The presented results of the effects of inhibitors on cell survival and growth (Figure 1A,B) are two different representations of the same data. The cells were treated with lapatinib, foretinib, or a mix of the two for 24 h (Figure 1) or 48 h (Figure A1) in the presence of EGF and HGF. The applied inhibitor concentrations were selected based on toxicity tests using a broad concentration range. The stimulation with both growth factors ensures that both signaling pathways initiated from EGFR and MET are activated, which mimics physiological conditions in the tumor micro milieu.

Both cell lines showed relative resistance to lapatinib (up to 10 µM). Foretinib reduced the percentage of viable cells in a dose-dependent manner (e.g., resulting in 50% cytotoxicity at 5 µM) while a combination of lapatinib and foretinib further decreased the number of viable cells (Figure 1A,B). At higher concentrations, mixed treatment with foretinib/lapatinib completely blocked the proliferation of examined cells. A proliferation value of below 1 was indicative of a toxic effect (Figure 1 and Figure A1). The application of Calcusyn software showed a synergistic interaction between both inhibitors (with a combination index (CI) < 1) at different concentration combinations in the two cell lines especially in the case of BT549 (Figure 1B,C). The inhibitory effect of combined treatment with lapatinib and foretinib was significantly enhanced compared to single-agent therapy in both cell lines (Figure 1 and Figure A1). These experiments indicate a dose-dependent synergistic interaction between foretinib and lapatinib in suppressing the growth and survival of triple-negative breast cancer cell lines.

### 2.2. Effects of EGFR and MET Inhibition on Downstream Signaling

**Figure 2:**
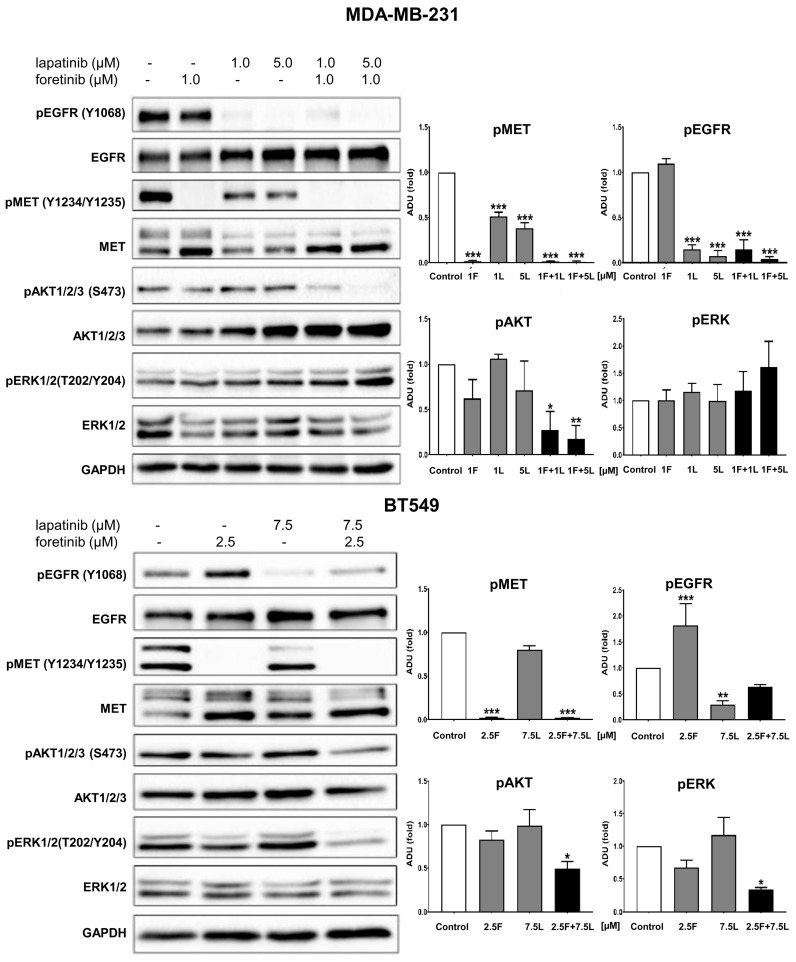
Activation of EGFR, MET, AKT, and ERK in inhibitor-treated TNBC cell lines. Representative immunoblots showing EGFR/pEGFR, MET/pMET, AKT/pAKT, and ERK/pERK levels in cellular extracts of control cells (incubated for 4 h only with 5 nM EGF and 30 ng/mL HGF) and cells treated with HGF, EGF, and the indicated concentrations of foretinib, lapatinib, or their combination. Graphs present densitometric analysis of protein bands for pEGFR, pMET, pERK, and pAKT. ADU stands for arbitrary densitometry units. The densitometry analysis for selected proteins was adjusted using the total protein content. The statistical significance was assessed versus the control. The significance level was set at *p* ≤ 0.05 (*), *p* ≤ 0.01 (**), or *p* ≤ 0.001 (***).

**Figure A2:**
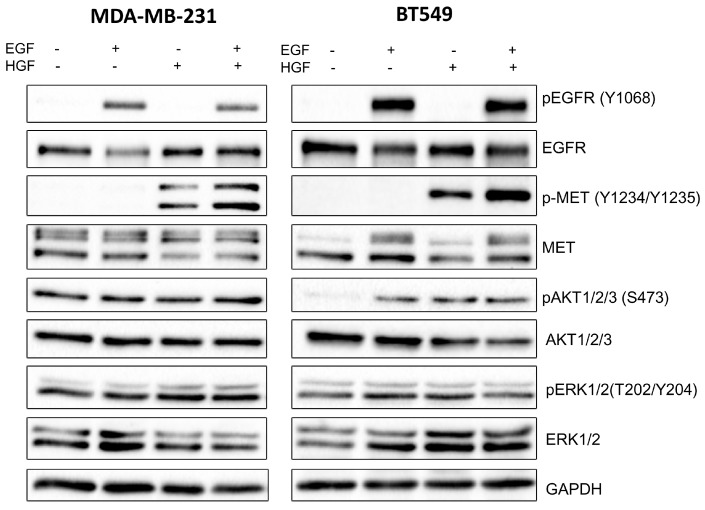
Activation of EGFR, MET, AKT, and ERK in TNBC cell lines. Representative immunoblots showing EGFR/pEGFR, MET/pMET, AKT/pAKT, ERK/pERK, and GAPDH levels in cellular extracts of non-stimulated cells as well as cells incubated for 4 h with 5 nM EGF, 30 ng/mL HGF, or their combination.

Given our interest in potential crosstalk, we studied the activation state of selected proteins involved in EGFR and MET signaling pathways in MDA-MB-231 and BT549 cells treated with combinations of inhibitors at non-toxic concentrations using Western blotting analysis (see Figure 1). In all tested conditions, cells were additionally stimulated with EGF and HGF. This resulted in a high level of phosphorylation of the functional cell surface receptors, EGFR (pY1068-level), and MET (pY1234/Y1235-levels), which is evident from the control sample in Figure 2 (other controls are shown in Figure A2). We investigated the changes in the receptor activation state and downstream signaling for both cell lines after treatment with drugs, alone or in combination. As expected, we observed that lapatinib was able to reduce the pEGFR level, and foretinib the pMET level in both cell lines. Of interest in MDA-MB-231, lapatinib (1 µM) also reduced the activation of the MET receptor (despite the presence of HGF). This is indicative of crosstalk and negative feedback in this cell line. Administration of lapatinib/foretinib simultaneously reduced the level of both phosphorylated receptors in both cell lines (Figure 2). At the tested non-toxic concentrations, each drug alone appeared insufficient to alter the activated phosphorylated levels of AKT (pAKT) or ERK (pERK), which are kinases reported to function in both signaling pathways. However, the combination of these two drugs at the applied concentration reduced the level of pAKT compared to control and single treatment conditions in both cell lines. This was most apparent in MDA-MB-231 cells. The level of pERK was reduced only in BT549 cells treated with the pair of inhibitors (Figure 2).

These results indicate that, when the inhibitors are used as monotherapy in the presence of HGF and EGF and at a concentration sufficient for the inhibitor to influence at least its own receptor phosphorylation, it is not translated into a reduction of survival and proliferative signaling. However, the combination of these EGFR and MET inhibitors blocked the activation of essential pathways required for cell growth and invasion (this is discussed below).

### 2.3. Influence of EGFR and MET Inhibition on the Cell Cycle of TNBC

**Figure 3:**
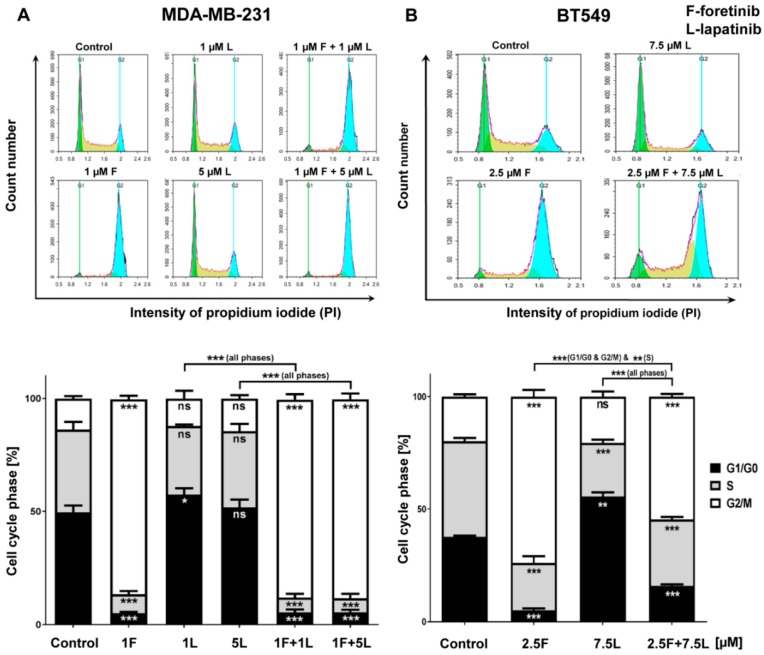
Effect of foretinib, lapatinib, or their combination on cell cycle phases in TNBC cell lines. Cell cycle distribution analysis was performed in (**A**) MDA-MB-231 and (**B**) BT549 cells that were untreated or treated with HGF, EGF, and the inhibitors at the indicated concentrations. Representative histograms as well as graphs are shown. The statistical significance was assessed versus the control. The significance level was set at *p* ≤ 0.05 (*), *p* ≤ 0.01 (**), or *p* ≤ 0.001 (***).

To better document the effect of foretinib, lapatinib, and their combination on cell growth, we evaluated the changes in the cell cycle 24 h after treatment of the cells with the drugs in the presence of EGF and HGF. As shown in Figure 3, lapatinib alone did not affect the distribution of cell populations in different phases of the cell cycle in the TNBC cell lines. However, treatment with foretinib notably induced the accumulation of cells in the G2/M phase (G2/M arrest), which was accompanied by a reduction of both the G1/G0 and S phases. This change in cell cycle distribution induced by foretinib was sustained in the cells that were treated with combinations of lapatinib and foretinib.

### 2.4. EGFR and MET Inhibition Affects Invadopodia and ECM Degradation Abilities of TNBC Cell Lines

**Figure 4:**
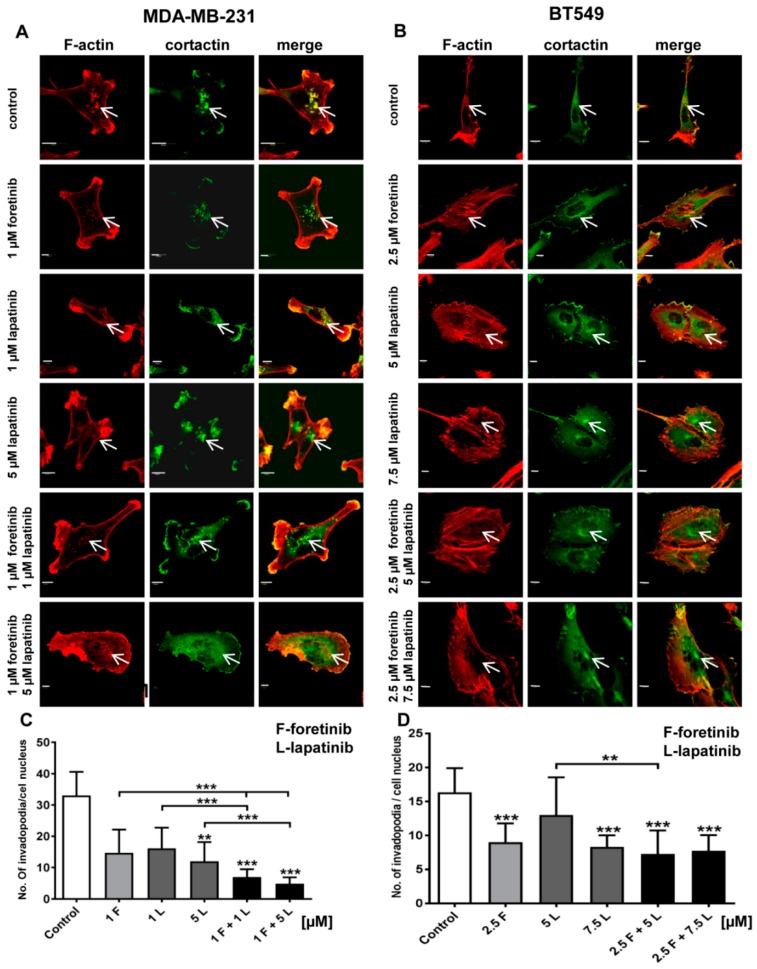
Influence of foretinib, lapatinib, and their combination on the subcellular organization of filamentous actin, the invadopodia marker cortactin, and the number of invadopodia in the (**A**) MDA-MB-231 and (**B**) BT549 cell lines. EGF and HGF-stimulated cells (control or treated with foretinib and lapatinib inhibitors, as indicated) on collagen type I-coated coverslips were stained for F-actin and cortactin. White arrows indicate invadopodia. Scale bar: 10 μm. Average number of invadopodia per cell nucleus in (**C**) MDA-MB-231 and (**D**) BT549 cells in control cells (treated only with EGF and HGF) and cells also treated with the indicated inhibitor concentrations. Invadopodia from 30 cells from three independent experiments were counted. Bars represent standard deviation. The statistical significance was assessed versus the control. The significance level was set at *p* ≤ 0.05 (*), *p* ≤ 0.01 (**), and *p* ≤ 0.001 (***).

Cancer cells form invadopodia to digest elements of the extracellular matrix and to create paths that can be used to invade through tissues [23]. Invadopodia are actin-rich adhesive structures with proteolytic activity, which are often formed by mesenchymally migrating cells. We knew from our earlier studies that MDA-MB-231 cells migrate in a mesenchymal way and are able to form invadopodia [24]. The second examined cell line, BT549, also forms invadopodia, owever they are smaller than those present in MDA-MB231 cells. We visualized the filamentous actin (F-actin) organization in drug-treated cells using phalloidin-Alexa Fluor 568. Additionally, we stained cells for cortactin, which is a marker of invadopodia. All cells were seeded on collagen-coated coverslips and were stimulated with EGF and HGF. Depending on the condition, they were also treated with foretinib, lapatinib, or their combination for 24 h. Incubation with foretinib or a combination of foretinib and lapatinib increased the cell area and appeared to generate cells with multiple nuclei (apparent from cortactin staining), which is seen in Figure 4.

This is in line with the effect of foretinib (or a combination of this drug with EGFR inhibitor) that was observed in the cell cycle analysis above. We demonstrated that cells treated with the inhibitors still form invadopodia based on colocalization of F-actin with cortactin (white arrows). However, when we quantified this effect, we observed a reduction in the number of invadopodia in inhibitor-treated cells compared to control cells (Figure 4). This suggests the negative feedback of inhibitors on invadopodia formation or stability.

**Figure 5:**
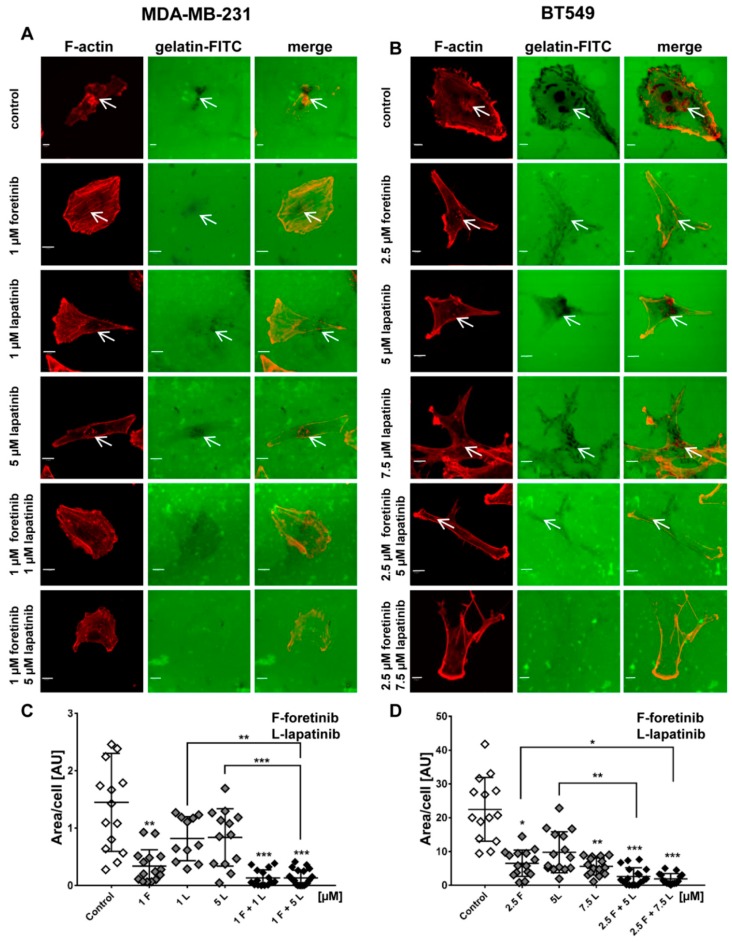
Influence of EGFR and MET inhibitors on the proteolytic activity of the (**A**) MDA-MB-231 and (**B**) BT549 cell lines. Cells were seeded on FITC-gelatin and after inhibitors treatment (12 h) they were fixed and stained for F-actin.Matrix degradation activity was identified by dark areas on the gelatin-FITC background. White arrows indicate invadopodia. Scale bar: 10 µM. The areas digested by (**C**) MDA-MB-231 and (**D**) BT549 cells were calculated using ImageJ software. Asterisks indicate differences between control and treated cells or between cells treated with different drugs. The significance level was set at *p* ≤ 0.05 (*), *p* ≤ 0.01 (**), or *p* ≤ 0.001 (***).

Next, we calculated the proteolytic activity of the invadopodia formed by cells in the different conditions (Figure 4C,D). In a gelatin-FITC degradation assay, sites of gelatin digestion appear as black spots on a fluorescent background. Control cells (stimulated with EGF and HGF) of both cell lines were able to digest gelatin strongly. Cells treated with a single inhibitor (especially foretinib) degraded the matrix less notably while cells administered with inhibitor combinations almost completely lost the ability to digest gelatin (Figure 5). These results suggest that the simultaneous blocking of signaling pathways connected to EGFR and MET receptors reduces the capability of cells to digest elements of extracellular matrix and, consequently, may also decrease their ability to invade tissues in a protease-dependent way.

### 2.5. Effects of Inhibitors on the Migration and Invasion Abilities of TNBC Cells

**Figure 6:**
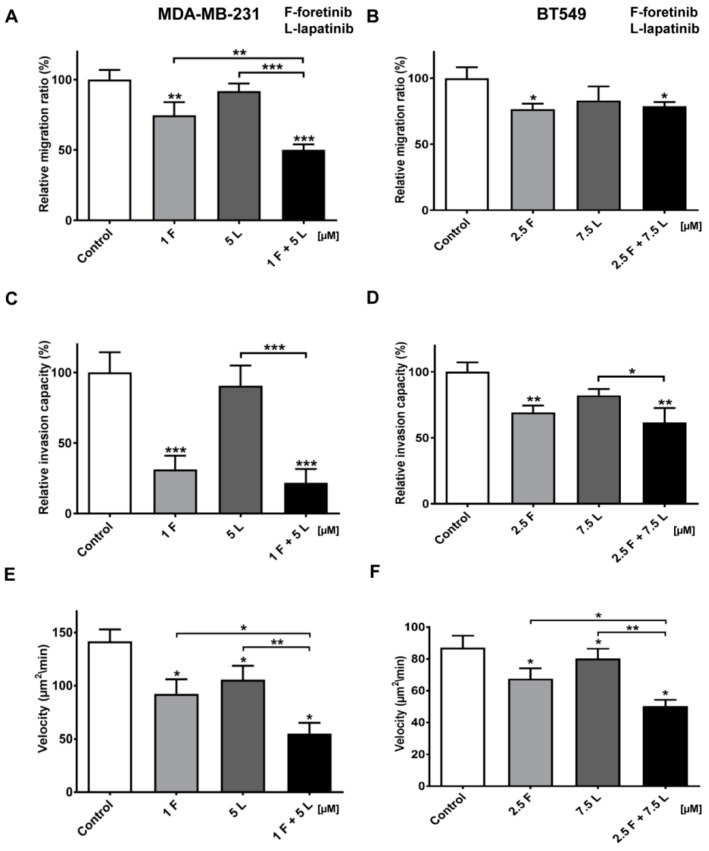
Migration and invasion capacities and invasion velocity of TNBC cells treated with MET and EGFR inhibitors. (**A**,**C**,**E**) MDA-MB-231 and (**B**,**D**,**F**) BT549 cells were incubated with foretinib (F) lapatinib (L), or their combination at the indicated concentrations (µM) for 24 h or 48 h. (**A**,**B**) Migration and (**C**,**D**) invasion experiments were conducted on Transwell filters, non-coated (migration), or coated with collagen type I (invasion). Results are expressed as the mean ± SD and are based on at least three independent experiments. Migration and invasion capacity in control cells is set to 100%. (**E**,**F**) Invasion velocity of control and treated cells. The change in area of the cell layer was continuously measured and the mean velocity derived from the area increase in time and was calculated (*n* = 8). The error bars represent SD. For all graphs, asterisks indicate conditions statistically significant, which is different from the control. The significance level was set at *p* ≤ 0.05 (*), *p* ≤ 0.01 (**), or *p* ≤ 0.001 (***).

**Figure A3:**
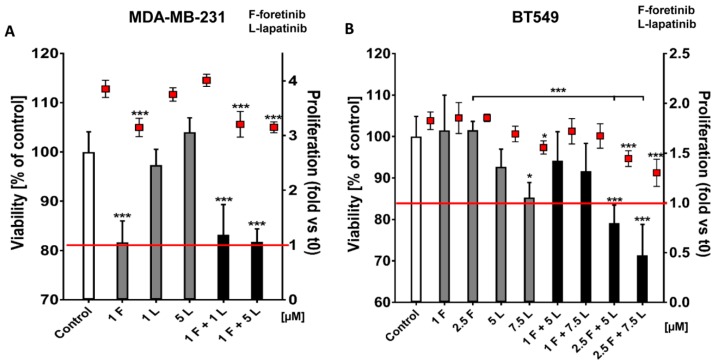
Influence of foretinib, lapatinib, and their combination on TNBC cell viability and proliferation in 3D conditions. Cell viability and proliferation of (**A**) MDA-MB-231 cells and (**B**) BT549 cells after 24 h of incubation with the indicated concentrations of inhibitors (in µM) were measured using XTT assay. Results expressed as the mean ± SD are representative for at least three independent experiments. The statistical significance was assessed versus the control. The significance level was set at *p* ≤ 0.05 (*), *p* ≤ 0.01 (**), or *p* ≤ 0.001 (***).

Since malignant cancer manifests itself via dissemination, we also investigated the combined effect of the inhibitors foretinib and lapatinib on the migration and invasion (i.e., migration in a 3D matrix) capacities of TNBC cells. Since we aimed to use concentrations that were not toxic or anti-proliferative, we retested the lower concentrations (1 µM foretinib or 1 and 5 µM lapatibnib) by using the XTT-assay in a 3D collagen context, which is similar to Reference [25]. Results shown in Figure A3 indicated that these concentrations were useful in evaluating the effects on migration and invasion. Initially, Boyden chamber migration assays were performed. After 24 h, a significant decrease in migration capacity was observed in cells treated with foretinib or a combination of foretinib/lapatinib for both cell lines in comparison to control cells (Figure 6A,B). The analogous results were obtained via an invasion assay in which cells invaded though a collagen gel on top of the membrane (Figure 6C,D). In MDA-MB-231 cells, the effect on migration and invasion was stronger in the case of cells treated with a combination of EGFR and MET inhibition compared to the use of single agents (Figure 6A,C). To strengthen the previous end-point experiment, we also measured cell invasion in a time-lapse experiment. Figure 6E,F show the velocity of the bulk cell population under the control and inhibitor-treated conditions. These data were obtained using a 3D wound healing-like invasion assay or cell-zone exclusion assay in which a confluent cell population embedded between collagen gel layers invaded a central cell-free collagen gel (ORIS, Platypus Technologies, [25,26]). The velocity was calculated using in-house-developed software tools (i.e., image-analysis using CELLMIA [27] and CellMissy [28]). Invasion velocity was significantly lower for the cells treated with inhibitors in comparison to the control cells. Furthermore, it was lower for cells treated with a combination of foretinib and lapatinib than for cells incubated with single agents, which confirms a synergistic effect of these drugs on the examined cells.

## 3. Discussion

To better characterize and understand different kinds of breast cancers, they were classified based on the presence of molecular markers. Luminal A and B subtypes exhibit expression of estrogen and/or progesterone receptors while the HER2 subtype demonstrates enrichment in HER2 expression with a lack of response to hormones. Triple-negative breast cancer is deficient in both hormone receptors and HER2 expression and shows high similarity to basal-like breast cancer (BLBC). Nevertheless, around 5% to 15% cases of BLBC are not triple-receptor-negative [1,12]. Despite constant furthering of our knowledge concerning the molecular background of this tumor, existing therapeutic strategies still fail to fully elicit a successful outcome. Currently used targeted therapies against breast cancer include the use of tamoxifen, fulvestrant, and aromatase inhibitors for ER-positive breast cancers and Herceptin and HER2 inhibitors for the HER2-positive breast cancer treatment [29]. In the case of TNBC, patients show initial responsiveness to conventional chemotherapy. However, they frequently experience a relapse that eventually leads to worse survival outcome compared to those with other breast cancer subtypes [12]. The lack of effective treatment following relapses and high metastasis rates also contribute to the bad prognosis of patients with TNBC [30].

A suggested new potential therapy directed against TNBC is the combined targeting of the growth factors receptors MET and EGFR. (1) Both are overexpressed simultaneously in this subtype [17]. (2) They activate numerous, partly overlapping, signaling pathways that promote cell growth, invasion, and cell survival with the predominant ones being Ras/ERK and PI3K/AKT pathways. (3) In a monotherapy context, they are frequently associated with therapeutic resistance to TKIs [2]. Moreover, in cancer development, their functional crosstalk has been reported with EGFR activating MET or, conversely, with MET expression regulating EGFR tyrosine phosphorylation and cell growth, e.g., in the presence of gefitinib (EGFR inhibitor) in SUM229 breast cancer cells [9]. Mueller et al. suggested that MET may mediate EGFR phosphorylation through co-association, which will lead to conformational changes in both receptors and regulation of the kinase activity of MET. This association of EGFR and MET and their mutual regulation suggest that inhibiting the kinase activity of these molecules individually may be insufficient. Thus, shifting from monotherapy to combination therapy, targeting both primary and rescue pathways, could be a promising approach. In non-small cell lung cancer cells, MET overexpression increased EGFR downstream signaling and the combined use of dual EGFR/HER2 and MET tyrosine kinase inhibitors resulted in a maximal growth inhibition [31]. Importantly, in view of therapy development, the MET and EGFR tyrosine kinases are actionable therapeutic targets due to their high expression in TNBC and several selective kinase inhibitors of which both are FDA approved for other cancers or in clinical trials [2].

In this study, we showed that two selected MET and EGFR inhibitors, foretinib and lapatinib, act effectively in the two TNBC cell lines. In a context of permanent ligand-based activation (as is occurring in the tumor), the inhibitors join forces to reduce several malignant properties of the TNBC cells including survival, AKT activation, cell growth, invadopodia formation, matrix proteolysis, and, importantly, migration and invasion. This is, to our knowledge, the first report showing that a combination of EGFR and MET inhibitors reduce not only TNBC cell viability but also cell invasion, which is connected to the devastating ability of these cells to form metastasis. The simultaneous usage of inhibitors of these two receptors led to not only better treatment efficiency but also and equally importantly prevented crosstalk between examined receptors.

Going into detail, we noticed that the combination of drugs inhibited cell viability in much stronger ways than the inhibitors when used independently. The average combination index was 0.68 for MDA-MB-231 cells and 0.28 for BT549 cells, which demonstrates the synergistic effect of foretinib and lapatinib (Figure 1). These results suggested that used inhibitors work collectively to decrease cell viability and proliferation in TNBC cells. Similar results were obtained by Kim et al. [17], Yi et al. [5], and Sohn et al. [3]. Our study also indicated that the pAKT level was decreased in both cell lines only when drugs were used in combination (Figure 2), which suggests that only a combination of EGFR and MET inhibitors is able to inhibit the downstream signaling pathways of these receptors. This result is in line with several recent studies, which have revealed that co-expression of RTKs can promote signaling crosstalk and maintain active Ras/ERK and PI3K/AKT signaling in the presence of kinase inhibitors to ensure cell survival. It was demonstrated that RTKs are co-expressed in 41 cancer cell lines [30] while ligands of RTKs may be able to overcome the drug sensitivity in “kinase-addicted” malignant cells. For example, the expression of HGF reduced sensitivity of several HER2-dependent breast cancer cell lines to lapatinib [2]. It was also shown that failure to induce inhibition of AKT can be a major cause of resistance to EGFR inhibitors [3]. In our research, the reduction in pEGFR level was connected to decreased pERK1/2 level only in one cell line: BT549. The fact that it is not decreased in the second cell line may be due to several reasons. First, in breast cancer cells, growth factors other than EGF and HGF may trigger the activation of the MAPK signaling pathway, which includes ERK1/2. Among these growth factors are insulin-like growth factor [2] or nerve growth factor [3]. Thus, even if EGFR is inhibited, other receptors of growth factors can lead to this pathway activation. Second, there may be mutations present in the genes encoding proteins present upstream of ERK1/2 in the MAPK signaling pathway, i.e., Raf or Ras. This may make these proteins constitutively active and, thus, lead to phosphorylation of ERK1/2 in the absence of an active EGFR receptor [4]. Third, it was shown that TNBC cell lines are characterized by a profile of gene expression similar to those of k-Ras or EGFR-mutant cancers and exhibit upregulation of pMEK and pERK [5,6]. All these reasons can cause the pERK level to remain unchanged despite the decreased level of pEGFR.

We also noticed that foretinib and its combination with lapatinib caused an accumulation of cells in the G2/M phase (G2/M arrest) (Figure 3). Additionally, we observed the appearance of many tetraploid cells after inhibitor treatment, which suggests that foretinib is able to impair the regulation of cell division. A similar effect was observed by Dufies et al. who showed that foretinib-treated chronic myelogenous leukemia cells exhibited increased size, spindle assembly checkpoint anomalies, and enhanced ploidy that collectively resulted in a mitotic catastrophe [32]. After foretinib and foretinib/lapatinib treatment, we also observed an increased cell size (Figure 4) and appearance of cells with an elevated number of nuclei, which confirms data obtained by flow cytometry. Both cell lines also formed a lower number of invadopodia after incubation with a combination of inhibitors (Figure 4). Additionally, we noticed that treatment with foretinib and lapatinib simultaneously reduced the ability of both types of cells to digest fluorescently-labeled gelatin (Figure 5). These processes were accompanied by a reduced level of pAKT. Our results are in line with data obtained by Yamaguchi et al. who indicated that active AKT is necessary for efficient invadopodia assembly. Overexpression of this kinase led to increased invadopodia formation and gelatin degradation [7]. Other researchers have also shown that elements of the PIK3/AKT signaling pathway, e.g., PDK1, are involved in active invadopodia assembly [8,9]. Under the treatment of the pair of inhibitors, migration and invasion abilities of examined cells were also reduced (Figure 6). The obtained data indicates that the combination of EGFR and MET inhibitors is able to reduce TNBC invasion more effectively than monotherapy.

In summary, the tested drug combination could be applied to overcome intrinsic EGFR resistance in TNBC. Furthermore, our studies support the findings of targeting multiple RTKs to prevent signaling crosstalk and resistance to kinase inhibitors. These results help to understand the RTK signaling in TNBC and indicate potential therapeutic strategies for patients with this type of cancer. These findings provide the rationale for testing foretinib in combination with lapatinib in clinical trials in TNBC. Potential biomarkers to identify patients that might benefit from this approach with high levels of EGFR and/or MET expression are still needed.

## 4. Materials and Methods

### 4.1. Chemicals

Rabbit polyclonal anti-cortactin antibodies were obtained from Santa Cruz Biotechnology (Dallas, TX, USA). Alexa Fluor 568-conjugated phalloidin, secondary anti-rabbit antibodies conjugated with Alexa Fluor 488, gelatin conjugated with FITC, fetal bovine serum (FBS), trypsin, glutamine, penicillin/streptomycin, and DMEM were purchased from Invitrogen (Carlsbad, CA, USA). Dako fluorescent mounting medium was obtained from Dako (Agilent, Santa Clara, CA, USA). The epidermal growth factor (EGF) and rat-tail collagen (acid-extracted, non-pepsin treated) type I were obtained from BD Biosciences (Agilent, Santa Clara, CA, USA). Cell Proliferation Kit II was purchased from Roche (Basel, Switzerland). Antibodies directed against EGFR (SC-03), MET (SC-10), AKT1/2/3 (SC-8312), and phospho-AKT1/2/3 (S473; SC-135651) were purchased from Santa Cruz Biotechnologies. Anti-phospho-EGFR (Y1069; catalogue no. 3777), anti-phospho-MET (Y1234/Y1235; no. 3077), anti-ERK1/2 (no. 9102), and anti-phospho-ERK1/2 (T202/Y204; no. 9101) antibodies were from Cell Signaling Technologies (Danvers, MA, USA). HGF was obtained from Sigma Aldrich (Saint Louis, MO, USA), foretinib from Santa Cruz Biotechnologies, and lapatinib from Selleckchem (Houston, TX, USA). All other chemicals were classified as analytical grade reagents.

### 4.2. Cell Culture

The human triple negative breast cancer cell lines, MDA-MB-231, and BT549 (both confirmed by LGC Cell Line Authentication Service) were grown in DMEM medium containing 10% FBS, 2 mM glutamine, and antibiotics (100 U/mL penicillin, 100 μg/mL streptomycin). The cells were cultured in 25 cm^2^ tissue culture flasks (Sarstedt, Nümbrecht, Germany)) at 37 °C in 5% CO_2_/95% humidified air and passaged twice a week using a 0.25% trypsin/0.05% EDTA solution.

### 4.3. Treatment of Cells with Inhibitors

The cells were incubated with the EGFR inhibitor lapatinib and the MET inhibitor foretinib separately or with a combination of both for 12, 24 or 48 h. The inhibitor concentrations used in the experiments were selected based on XTT experiments and matched to the sensitivity of a given cell line. For each assay, cells were stimulated with 5 nM EGF and 30 ng/mL HGF to imitate conditions present in the breast cancer microenvironment. Cells incubated only with growth factors and 0.1% DMSO (inhibitors solvent) were used as a control.

### 4.4. Collagen Matrix Preparation

The solution for collagen matrix preparation consisted of type I collagen (2 mg/mL, final concentration), MEM, and 8.3 mM NaHCO_3_ in Hank’s Balanced Salt Solution and its pH was adjusted to a pH of 7.4–8 with 1 M NaOH.

### 4.5. Western Blot Analysis 

Cells were lysed in urea-containing buffer and were supplemented with protease and phosphatase inhibitor cocktails (Sigma Aldrich). The protein concentration of the cell lysates was determined by a standard Bradford procedure [33]. Additionally, 24 h after seeding in 60 mm culture dishes, cells (1 × 10^6^) were treated for 4 h with 1 to 2.5 μM of foretinib, 1 to 7.5 μM of lapatinib, or a combination of the two in medium containing 30 ng/mL HGF and 5 nM EGF. Samples were analyzed by SDS-PAGE, according to the procedure described by Laemmli [34], and transferred to nitrocellulose sheets, according to Towbin et al [35]. Anti-MET, anti-p-MET, anti-EGFR, anti-pEGFR, anti-AKT1/2/3, anti-pAKT1/2/3, anti-ERK 1/2, and anti-pERK 1/2 were used as primary antibodies. Goat anti-mouse or goat anti-rabbit antibody conjugated to horseradish peroxidase (HRP) was applied according to the manufacturer’s protocols. Immunoblots were developed using the Luminol Reagent (Bio-rad, Hercules, CA, USA) and scanned by using the ChemiDoc gel scanner (Bio-Rad). Quantitative analysis was performed by using Image Lab software (Bio-Rad). Bands for phosphorylated proteins were normalized to total protein content (Ponceau S staining) and compared to control cells. All experiments were done in triplicate.

### 4.6. Cell Cytotoxicity and Proliferation Assay in 2D and 3D

The Cell Proliferation Kit II (XTT) (Roche, Hercules, CA, USA) was used according to the manufacturer’s protocol. The XTT labeling mixture was added in parallel samples at time 0 (t0) and after 24 or 48 h of cell growth in the absence or presence of inhibitors (foretinib, lapatinib, or their combination) at the indicated concentrations. For both 2D and 3D matrix-embedded cultures, absorbance was measured three hours after XTT addition. All conditions were prepared in four replicates. The exact protocol of seeding cells in 2D and 3D conditions, the execution of the test, and the calculation of cytotoxicity and the proliferation rate was described earlier by Huyck et al. (2012).

### 4.7. Calculation of the Combination Index

Combination analysis of treatment with foretinib and lapatinib was performed using the Calcusyn software program (Biosoft, Cambridge, UK), which calculates a combination index, according to Chou and Talalay-derived equations [36]. CI <1, =1, and >1 represent synergistic, additive, and antagonist effects, respectively.

### 4.8. Cell Cycle Analysis

BT549 (3.5 × 10^5^) and MDA-MB-231 (2.5 × 10^5^) cells were plated on six-well plates. After 24 h, the medium was replaced with a medium containing growth factors (5 nM EGF and 30 ng/mL HGF) in combination with either DMSO (0.1% final concentration, mock treatment), foretinib (1–2.5 μM), lapatinib (1–7.5 μM), or a combination of the two inhibitors. Cells were incubated in the presence of these compounds for 24 h. Afterwards, the medium was discarded, cells were washed with PBS without Ca^2+^/Mg^2+^, trypsinized, centrifuged (100× *g*, 5 min), and fixed with ice-cold 70% ethanol for at least 24 h in −20 °C. Then, cells were washed three times with PBS (800× *g*, 5 min), incubated with RNase A (final concentration 10 μg/mL, 45 min, room temperature), stained with propidium iodide (final concentration 50 μg/mL, 30 min, 4 °C), and subsequently analyzed with a NovoCyte flow cytometer and ACEA NovoExpress software (ver. 1.2.4; ACEA Biosciences, San Diego, CA, USA).

### 4.9. Immunofluorescence

The subcellular distribution of actin filaments and cortactin was examined by immunofluorescence. Cells (3 × 10^4^ per coverslip) were seeded on collagen type I-coated coverslips in 24-well plates and grown for 24 h. To obtain a coating of collagen, prepared as described in the collagen matrix preparation section, the coverslips were incubated for 30 min at 37 °C and 5% CO_2_. Next, the cells were fixed with 4% formaldehyde for 20 min at room temperature and permeabilized with 0.1% Triton X-100 in PBS for 5 min. Coverslips were blocked for 30 min with 1% bovine serum albumin in PBS. Anti-cortactin antibodies, followed by Alexa Fluor 488-conjugated anti-rabbit secondary antibodies, were applied to visualize this protein. Actin filaments were stained with Alexa Fluor 568-labeled phalloidin. After incubation and washing steps, coverslips were mounted with Dako fluorescent mounting medium. For each condition, about 25 cells were imaged (Olympus FV500 confocal laser scanning microscope, Tokio, Japan) in three independent experiments and representative cells are shown. Quantitative analysis of the number of invadopodia per nuclei was performed using ImageJ software. Only invadopodia positive for F-actin and cortactin were scored and 30 cells were analyzed per condition.

### 4.10. Fluorescent-Gelatin Degradation Assay

The test was conducted according to the procedure described by Artym [37]. Poly-L-lysine-coated coverslips were washed with PBS and fixed with 0.5% glutaraldehyde for 15 min, which was followed by extensive washing. Afterward, the coverslips were inverted on an 80 μL drop of gelatin conjugated with FITC and incubated for 10 min at room temperature. After washing with PBS, the residual reactive groups were quenched with 5 mg/mL sodium borohydride for 1 min followed by extensive washing with PBS. Then, 4 × 10^4^ cells were plated in 24-well plates containing a coverslip coated with fluorescent gelatin matrix and incubated in the presence of foretinib, lapatinib, or a combination at 37 °C. After 12 h, cells were fixed with 4% formaldehyde in PBS for 20 min and permeabilized for 5 min with 0.5% Triton X-100. Cells were stained for filamentous actin. Confocal images were collected using an Olympus FV500 confocal laser scanning microscope. The sites of degraded matrix were visible as dark areas (spots) in the bright fluorescent gelatin matrix. The area of gelatin digestion was calculated for 15 cells per condition using ImageJ software [38].

### 4.11. Migration Assay

Cell migration tests were performed using Transwell™ filters (BD Biosciences, Franklin Lakes, NJ, USA) in a 24-well plate. Prior to the assay, cells were starved for 6 h in serum-free DMEM medium. The lower compartment of the Transwell™ contained 500 μL of medium consisting of 80% DMEM, 20% FBS, 5 nM EGF, and 30 ng/mL of HGF. Cells (5 × 10^4^) were seeded onto a rehydrated Transwell™ insert in medium without FBS in the absence (control) or presence of foretinib, lapatinib, or foretinib/lapatinib. After 24 h, non-migrating cells on the upper side of the filters were removed. Cells that migrated through the membrane were fixed with 4% formaldehyde, stained with Hoechst 33342 (Invitrogen), and counted under a fluorescent microscope Olympus FV500 (Tokio, Japan). The results are showed as a relative migration factor (%) where the number of control cells that migrated through the Transwell™ filters are presented as 100%. The experiments were performed three times. Each independent experiment consisted of three measurements.

### 4.12. Invasion Assay

MDA-MB-231 and BT549 cells (5 × 10^4^) were seeded after 6 h of starvation in serum-free DMEM onto Transwell™ filters coated with a collagen gel (1 mg/mL) and prepared as described in the collagen matrix preparation section. Medium containing 20% fetal bovine serum with EGF and HGF was present in the lower compartment as a chemoattractant. After 24 h, non-invading cells and collagen on the upper side of the filters were removed. Cells that had migrated through the membrane were fixed with 4% formaldehyde, stained with Hoechst 33342, and counted under a fluorescent microscope Olympus FV500 (Tokio, Japan). The results are presented as a relative invasion factor (%) versus the control. The number of control cells that invaded through the Transwell™ filters is set as 100%.

### 4.13. Timelapse Invasion Assay 

The 3D collagen matrix was prepared as described in the collagen coating preparation section. 4.5 × 10^3^ cells were seeded in 96-well plates on top of a thin layer of collagen gel and around a shielded central zone in the middle of the well, according to the ORIS cell invasion protocol [27]. The cells and the central cell-free zone were covered with a second collagen gel layer (40 µL volume). After polymerization, 100 μL of growth medium was added on top of the 3D collagen I gel containing the cell layer. Phase-contrast time-lapse movies were recorded with a time interval of 20 min using a 10 × UPlanFL objective (N.A. 0.30) on a CellM system with an IX81 microscope (Olympus). Control cells and cells treated with inhibitors were allowed to invade the central zone for 48 h. Image and data analyses were done using CELLMIA image processing software [27] and the data management and analysis software CellMissy [28], respectively. The cell invasion velocity is based on the increase in areas covered by the cells in a given time.

### 4.14. Statistical Analysis

All data are given as means ± SD. Their significance was determined with GraphPad Prism 7 Software using one-way ANOVA followed by the Tukey test (i.e., cytotoxicity and proliferation assay, cell cycle analysis, and migration and invasion assays) or the Kruskal–Wallis method, which was followed by the Dunn’s post-hoc test (i.e., number of invadopodia and FITC-gelatin digestion).

## 5. Conclusions

Our data reveals that a combination of foretinib and lapatinib effectively reduced the viability and proliferation of the examined cell lines. Moreover, both inhibitors decreased the number of invadopodia formed by TNBC cells and their migration/invasion capacity. Thus, the specific targeting of the EGFR and c-Met may be a useful strategy against triple negative breast cancer.

## References

[1] Foulkes W.D., Smith I.E., Reis-Filho J.S. (2010). Triple-Negative Breast Cancer. N. Engl. J. Med..

[2] Linklater E.S., Tovar E.A., Essenburg C.J., Turner L., Madaj Z., Winn M.E., Melnik M.K., Korkaya H., Maroun C.R., Christensen J.G. (2016). Targeting MET and EGFR crosstalk signaling in triple-negative breast cancers. Oncotarget.

[3] Sohn J., Liu S., Parinyanitikul N., Lee J., Hort obagyi G.N., Mills G.B., Ueno N.T., Gonzalez-Angulo A.M. (2014). cMET activation and EGFR-directed therapy resistance in triple-negative breast cancer. J. Cancer.

[4] Burness M.L., Grushko T.A., Olopade O.I. (2010). Epidermal growth factor receptor in triple-negative and basal-like breast cancer: Promising clinical target or only a marker?. Cancer J..

[5] Yi Y.W., You K., Bae E.J., Kwak S.J., Seong Y.S., Bae I. (2015). Dual inhibition of EGFR and MET induces synthetic lethality in triple-negative breast cancer cells through downregulation of ribosomal protein S6. Int. J. Oncol..

[6] Viale G., Rotmensz N., Maisonneuve P., Bottiglieri L., Montagna E., Luini A., Veronesi P., Intra M., Torrisi R., Cardillo A. (2009). Invasive ductal carcinoma of the breast with the “triple-negative” phenotype: Prognostic implications of EGFR immunoreactivity. Breast Cancer Res. Treat..

[7] Bhargava R., Gerald W.L., Li A.R., Pan Q., Lal P., Ladanyi M., Chen B. (2005). EGFR gene amplification in breast cancer: Correlation with epidermal growth factor receptor mRNA and protein expression and HER-2 status and absence of EGFR-activating mutations. Mod. Pathol..

[8] Jorissen R.N., Walker F., Pouliot N., Garrett T.P.J., Ward C.W., Burgess A.W. (2003). Epidermal growth factor receptor: Mechanisms of activation and signalling. Exp. Cell Res..

[9] Mueller K.L., Yang Z.Q., Haddad R., Ethier S.P., Boerner J.L. (2010). EGFR/Met association regulates EGFR TKI resistance in breast cancer. J. Mol. Signal.

[10] Eccles S. (2011). The epidermal growth factor receptor/Erb-B/HER family in normal and malignant breast biology. Int. J. Dev. Biol..

[11] Toi M., Osaki A., Yamada H., Toge T. (1991). Epidermal growth factor receptor expression as a prognostic indicator in breast cancer. Eur. J. Cancer.

[12] Hsu Y.H., Yao J., Chan L.C., Wu T.J., Hsu J.L., Fang Y.F., Wei Y., Wu Y., Huang W.C., Liu C.L. (2014). Definition of PKC-α, CDK6, and MET as therapeutic targets in triple-negative breast cancer. Cancer Res..

[13] Raghav K.P., Wang W., Liu S., Chavez-MacGregor M., Meng X., Hortobagyi G.N., Mills G.B., Meric-Bernstam F., Blumenschein G.R., Gonzalez-Angulo A.M. (2012). cMET and phospho-cMET protein levels in breast cancers and survival outcomes. Clin. Cancer Res..

[14] Birchmeier C., Birchmeier W., Gherardi E., Vande Woude G.F. (2003). Met, metastasis, motility and more. Nat. Rev. Mol. Cell Biol..

[15] Lai A.Z., Abella J.V., Park M. (2009). Crosstalk in Met receptor oncogenesis. Trends Cell Biol..

[16] Ortiz-Zapater E., Lee R.W., Owen W., Weitsman G., Fruhwirth G., Dunn R.G., Neat M.J., McCaughan F., Parker P., Ng T. (2017). MET-EGFR dimerization in lung adenocarcinoma is dependent on EGFR mtations and altered by MET kinase inhibition. PLoS ONE.

[17] Kim Y.J., Choi J.S., Seo J., Song J.Y., Eun Lee S., Kwon M.J., Kwon M.J., Kundu J., Jung K., Oh E. (2014). La MET is a potential target for use in combination therapy with EGFR inhibition in triple-negative/basal-like breast cancer. Int. J. Cancer.

[18] Comoglio P.M., Giordano S., Trusolino L. (2008). Drug development of MET inhibitors: Targeting oncogene addiction and expedience. Nat. Rev. Drug Discov..

[19] Engelman J.A., Zejnullahu K., Mitsudomi T., Song Y., Hyland C., Park J.O., Lindeman N., Gale C.-M., Zhao X., Christensen J. (2007). MET Amplification Leads to Gefitinib Resistance in Lung Cancer by Activating ERBB3 Signaling. Science.

[20] Stommel J.M., Kimmelman A.C., Ying H., Nabioullin R., Ponugoti A.H., Wiedemeyer R., Stegh A.H., Bradner J.E., Ligon K.L., Brennan C. (2007). Coactivation of Receptor Tyrosine Kinases Affects the Response of Tumor Cells to Targeted Therapies. Science.

[21] Guo A., Villen J., Kornhauser J., Lee K.A., Stokes M.P., Rikova K., Possemato A., Nardone J., Innocenti G., Wetzel R. (2008). Signaling networks assembled by oncogenic EGFR and c-Met. Proc. Natl. Acad. Sci. USA.

[22] Turke A.B., Zejnullahu K., Wu Y.L., Song Y., Dias-Santagata D., Lifshits E., Toschi L., Rogers A., Mok T., Sequist L. (2010). Preexistence and clonal selection of MET amplification in EGFR mutant NSCLC. Cancer Cell.

[23] Gimona M., Buccione R., Courtneidge S.A., Linder S. (2008). Assembly and biological role of podosomes and invadopodia. Curr. Opin. Cell Biol..

[24] Simiczyjew A., Mazur A.J., Ampe C., Malicka-Błaszkiewicz M., Van Troys M., Nowak D. (2015). Active invadopodia of mesenchymally migrating cancer cells contain both β and γ cytoplasmic actin isoforms. Exp. Cell Res..

[25] Huyck L., Ampe C., Van Troys M. (2012). The XTT cell proliferation assay applied to cell layers embedded in three-dimensional matrix. Assay Drug Dev. Technol..

[26] Hulkower K.I., Herber R.L. (2011). Cell migration and invasion assays as tools for drug discovery. Pharmaceutics.

[27] Van Troys M., Masuzzo P., Huyck L., Bakkali K., Waterschoot D., Martens L., Ampe C. (2018). Analysis of invasion dynamics of matrix-embedded cells in a multi-sample format. Methods Mol. Biol..

[28] Masuzzo P., Hulstaert N., Huyck L., Ampe C., Van Troys M., Martens L. (2013). CellMissy: A tool for management, storage and analysis of cell migration data produced in wound healing-like assays. Bioinformatics.

[29] Lukong K.E. (2017). Understanding breast cancer—The long and winding road. BBA Clin..

[30] Wilson T.R., Fridlyand J., Yan Y., Penuel E., Burton L., Chan E., Peng J., Lin E., Wang Y., Sosman J. (2012). Widespread potential for growth-factor-driven resistance to anticancer kinase inhibitors. Nature.

[31] Agarwal S., Zerillo C., Kolmakova J., Christensen J.G., Harris L.N., Rimm D.L., DiGiovanna M.P., Stern D.F. (2009). Association of constitutively activated hepatocyte growth factor receptor (Met) with resistance to a dual EGFR/Her2 inhibitor in non-small-cell lung cancer cells. Br. J. Cancer.

[32] Dufies M., Jacquel A., Robert G., Cluzeau T., Puissant A., Fenouille N., Legros L., Raynaud S., Cassuto J.P., Luciano F. (2011). Mechanism of action of the multikinase inhibitor Foretinib. Cell Cycle.

[33] Bradford M.M. (1976). A rapid and sensitive method for the quantitation of microgram quantities of protein utilizing the principle of protein-dye binding. Anal. Biochem..

[34] Laemmli U.K. (1970). Cleavage of structural proteins during the assembly of the head of bacteriophage T4. Nature.

[35] Towbin H., Staehelin T., Gordon J. (1979). Electrophoretic transfer of proteins from polyacrylamide gels to nitrocellulose sheets: Procedure and some applications. Proc. Natl. Acad. Sci. USA.

[36] Chou T.C., Talalay P. (1984). Quantitative analysis of dose-effect relationships: The combined effects of multiple drugs or enzyme inhibitors. Adv. Enzyme Regul..

[37] Artym V.V., Zhang Y., Seillier-Moiseiwitsch F., Yamada K.M., Mueller S.C. (2006). Dynamic interactions of cortactin and membrane type 1 matrix metalloproteinase at invadopodia: Defining the stages of invadopodia formation and function. Cancer Res..

[38] Schneider C.A., Rasband W.S., Eliceiri K.W. (2012). NIH Image to ImageJ: 25 years of image analysis. Nat. Methods.

